# Supplementary material for: Impact of three weeks of integrative neuromuscular training on the athletic performance of elite female boxers
Source: PeerJ. 2024 Oct 30;12:e18311. doi: 10.7717/peerj.18311 (PMC11531254; doi:10.7717/peerj.18311)
Supplement: Supplemental Information 1 [file peerj-12-18311-s001.docx]

**Table S1** Basic strength Training

| **Exercise** | **Load** | **Sets** | **Time/ Repetition** | **Recovery（min）** |
| --- | --- | --- | --- | --- |
| Barbell bench press | 30%-90%1RM | 6 | 3-8rpes | 1min |
| Barbell pull | 30%-90%1RM | 6 | 3-8rpes | 1min |
| Barbell squat | 30%-90%1RM | 6 | 3-8rpes | 1min |
| Barbell hip punch | 30%-90%1RM | 6 | 3-8rpes | 1min |

**Table S2** Plyometric training

| **Exercise** | **Load** | **Sets** | **Repetition** | **Recovery（min）** |
| --- | --- | --- | --- | --- |
| Barbell bench press+Barbell piece flat bench press | 85%1RM+10kg/5kg | 5 | 5+10 | 2min |
| Barbell squat+Jump with elastic band | 85%1RM+elastic band | 5 | 5+6 | 2min |
| Barbell high pull+jumping box | 85%1RM+Jump box is 85% or more of the maximum vertical jump height | 5 | 5+6 | 2min |
| Barbell barrel squat thrust +Bulgarian bags down. | 85%1RM+Bulgarian Bags | 5 | 5+5 | 2min |
| Barbell lunge squat+Lunge and alternate jumps | 85%1RM+Own weight | 5 | 5+6 | 2min |

**Table S3** Explosive Power Training

| **Exercise** | **Load** | **Sets** | **Repetition** | **Recovery（min）** |
| --- | --- | --- | --- | --- |
| Hanging high pull with a barbell | 60-70%1RM | 3 | 6 | 2min |
| Barbell pull (explosion) | 60-70%1RM | 3 | 6 | 2min |
| Dumbbell single-arm snatch | 60-70%1RM | 3 | 6 | 2min |
| Barbell cannonball single arm front push | 60-70%1RM | 3 | 6 | 2min |
| Barbell bench press (explosion) | 60-70%1RM | 3 | 6 | 2min |
| Barbell barrel squat thrust | 60-70%1RM | 3 | 6 | 2min |
| Barbell hip punch | 60-70%1RM | 3 | 6 | 2min |

**Table S4** High Intensity Interval Training (HIIT)

| **Exercise** | **Load** | **Sets** | **Time or Repetition** | **Recovery（s）** |
| --- | --- | --- | --- | --- |
| Barbell pad push-up | 10kg、5kg barbell | 3 | 30s | 15s |
| Dumbbell Rapid Air Punch | 2.5kg、1kg dumbbell | 3 | 30s | 15s |
| kettlebell lift | 10kg、6kg kettlebell | 3 | 30s | 15s |
| Elastic band-assisted jumping | elastic band | 3 | 30s | 15s |
| Barbell cannon rotational push | 10kg,5kg | 3 | 30s | 15s |
| ViPR moves left and right | 8kg,4kg | 3 | 30s | 15s |
| Small hurdle obstacle jump | own weight | 3 | 30s | 15s |
| Battle Rope | Weight of the rope | 3 | 30s | 15s |
| Climbing machine | own weight | 3 | 30s | 15s |

ViPR:Vitality Performance Re-conditioning

**Table S5** Speed Interval Training

| **Exercise** | **Load** | **Sets** | **Repetition** | **Recovery（s）** |
| --- | --- | --- | --- | --- |
| 6s short sprint | - | 9 | 1 | 54s |

**Table S6** Energy metabolism training

| **Exercise** | **Load** | **Sets** | **Repetition** | **Recovery（min）** |
| --- | --- | --- | --- | --- |
| 3000m run | 150-160 HR_max_ | 2 | 1 | 3min |

**Table S7** Functional Training 1 (Barbell)

| **Exercise** | **Load** | **Sets** | **Repetition** | **Recovery（s）** |
| --- | --- | --- | --- | --- |
| Barbell piece squat unilateral push-up conversion | 10kg,5kg | 1 | 50 | 30s |
| Barbell Piece Straight Arm Rings | 10kg,5kg | 1 | 50 | 30s |
| Barbell piece unilateral stirrup rotation | 10kg,5kg | 1 | 50 | 30s |
| Barbell Piece Fast Forward Push | 10kg,5kg | 1 | 50 | 30s |
| Single leg hard pull explosive front jump | 10kg,5kg | 1 | 50 | 30s |
| Barbell push-up | 10kg,5kg | 1 | 50 | 30s |
| Barbell Piece Forward Push Turning Jumps | 10kg,5kg | 1 | 50 | 30s |
| Barbell piece head wraps around ring | 10kg,5kg | 1 | 50 | 30s |

**Table S8** Functional Training 2 (Kettlebell)

| **Exercise** | **Load** | **Sets** | **Repetitions** | **Recovery（s）** |
| --- | --- | --- | --- | --- |
| kettlebell swing | 50 | 1 | 50 | 30s |
| kettlebell swing forward jump | 50 | 1 | 50 | 30s |
| kettlebell swing backward jump | 50 | 1 | 50 | 30s |
| Kettlebell one-arm stirrup swing right side | 50 | 1 | 50 | 30s |
| Kettlebell one-arm stirrup swing left side | 50 | 1 | 50 | 30s |
| Kettlebell left and right turn U-swing | 50 | 1 | 50 | 30s |
| kettlebell jerk | 50 | 1 | 50 | 30s |
| kettlebell swing spinning jump | 50 | 1 | 50 | 30s |

**Table S9** Functional Training 3 (ViPR)

| **Exercise** | **Load** | **Sets** | **Repetition** | **Recovery（min）** |
| --- | --- | --- | --- | --- |
| ViPR Left and Right Movement | 30 | 1 | 30 | 1min |
| ViPR uppercut | 30 | 1 | 30 | 1min |
| ViPR hook | 30 | 1 | 30 | 1min |
| Barbell Cannon Ski Step Side Insertion | 30 | 1 | 30 | 1min |
| ViPR Lunge Left-Right Slanting Downward Chop | 30 | 1 | 30 | 1min |

**Table S10** Agility training

| **Exercise** | **Load** | **Sets** | **Repetition** | **Recovery（s）** |
| --- | --- | --- | --- | --- |
| hexagonal jump | 160-190 HR_max_ | 3 | 1 | 15s |
| hurdle rack | 160-190 HR_max_ | 3 | 1 | 15s |
| Shadow Exercise | 160-190 HR_max_ | 3 | 1 | 15s |
| rope ladder | 160-190 HR_max_ | 3 | 1 | 15s |
| jump forward and backward | 160-190 HR_max_ | 3 | 1 | 15s |
| Sliding jumps on both sides | 160-190 HR_max_ | 3 | 1 | 15s |
| Move left and right to change legs to jump up and down | 160-190 HR_max_ | 3 | 1 | 15s |
| run in all directions | 160-190 HR_max_ | 3 | 1 | 15s |

**Table S11** Core Stability Training

| **Exercise** | **Load** | **Sets** | **Repetition** | **Recovery（s）** |
| --- | --- | --- | --- | --- |
| Russia twist with medicine ball | 30 times | 2 | 30 times | 15s |
| Side bridge | 1 min | 2 | 1 min | 15s |
| Glute bridge | 1 min | 3 | 1 min | 15s |
| One leg stance on Bosu ball with eyes closed | 30s | 3 | 30s | 15s |
| Opposite side arm and leg raise on Bosu ball | 30s | 2 | 30s | 15s |
| Tennis ball receiving with one leg on Bosu ball | 30s | 2 | 30s | 15s |
